# Changes in spatial bodily pain distribution one year after benign hysterectomy with emphasis on prevalence and risk factors for de novo and persistent pelvic pain- a prospective longitudinal multicenter study

**DOI:** 10.1186/s12905-024-03474-5

**Published:** 2024-12-20

**Authors:** Peter Lukas, Lena Nilsson, Ninnie Borendal Wodlin, Lars Arendt-Nielsen, Preben Kjølhede

**Affiliations:** 1 Department of Obstetrics and Gynecology in Linköping, and Department of Biomedical and Clinical Sciences, Linköping University, Linköping, S-581 85 Sweden; 2 Department of Anesthesiology and Intensive Care in Linköping, and Department of Biomedical and Clinical Sciences, Linköping University, Linköping, S-581 85 Sweden; 3 Center for Neuroplasticity and Pain (CNAP), Department of Health Science and Technology, Faculty of Medicine, Aalborg University, Aalborg, Denmark; 4 Department of Medical Gastroenterology, Mech-Sense, Aalborg University Hospital, Aalborg, Denmark; 5 Steno Diabetes Center North Denmark, Clinical Institute, Aalborg University Hospital, Aalborg, Denmark

**Keywords:** Hysterectomy, Pain thresholds, Pelvic pain, Risk factors, Spread of bodily pain

## Abstract

**Background:**

The objectives were to determine the prevalence of de novo and persistent pelvic pain after benign hysterectomy and to assess risk factors.

**Methods:**

A Swedish prospective multicenter study of 440 women undergoing benign hysterectomy was conducted between October 2011 and March 2017. Measures of pain, the spatial extent of bodily pain, and pain sensitivity were assessed using a self-reporting questionnaire, Margolis’s patient pain drawing, and quantitative sensory testing of pain thresholds for pressure, heat, and cold, respectively. Quality of life was evaluated by EQ-5D-3L and SF-36. Psychological distress was assessed by the Hospital Anxiety and Depression Scaleand the Stress-Coping Inventory. Logistic regression models were used to assess risk factors, and the outcome was presented as an adjusted odds ratio (aOR) and 95% confidence interval (CI).

**Results:**

Preoperatively, 18.0% of the women reported no bodily pain, 41.5% had pelvic pain, either as the only location (7.0%) or along with pain in other locations (34.5%), and 40.5% had non-pelvic pain only. Postoperatively, 6.2% developed de novo pelvic pain and 16.4% had persistent pelvic pain. De novo pelvic pain developed exclusively in women who preoperatively had non-pelvic pain only. Risk factors for de novo pelvic pain were a long hospital stay (aOR 1.50 (95%CI) 1.02–2.21)), high preoperative pain intensity (aOR 1.25 (95%CI 1.01–1.62)) and a high number of pain areas (aOR 1.15 (95%CI 1.05–1.27)), along with anxiety (aOR 10.61 (95%CI 1.84–61.03)) and low EQ-5D-3L health index (aOR 0.02 (95%CI 0.00–0.31)). Risk factors for persistent pelvic pain were lower age (aOR 0.89 (95%CI 0.81–0.97)), higher number of pain areas (aOR 1.08 (95%CI 1.02–1.14)), and a higher frequency of preoperative pain (aOR 12.75 (95%CI 2.24–72.66)).

**Conclusion:**

Although hysterectomy appeared to be reasonably effective in curing pelvic pain, a non-negligible proportion of women developed de novo pelvic pain or had persistent pelvic pain. De novo pelvic pain seemed to affect only those who preoperatively had widespread bodily pain. Women at risk for de novo and persistent pelvic pain after hysterectomy could be identified preoperatively.

**Trial registrations:**

The study was retrospectively registered in ClinicalTrial.gov (NCT01526668) on 01/27//2012.

## Introduction

The overall purpose of benign hysterectomy is generally to improve the health-related quality of life. The long-term outcome is therefore of particular importance when evaluating the effect of surgery. Postoperative pain can be a problem by delaying the patient’s recovery and causing long-term discomfort. The prevalence of chronic pelvic pain after benign hysterectomy varies in the range of 5–32% [1]. The underlying mechanisms of chronic pain after surgery are not fully understood, although several risk factors have been identified. In connection with benign hysterectomy, risk factors are reported to include open surgery, psychological factors such as depression, anxiety, pain catastrophizing, and pain problems elsewhere, and severe acute postoperative pain intensity [2–11]. Furthermore, preoperative pain seems to play a significant role in the development of chronic pain after surgery [1, 10]. The studies published so far conclude that preexisting pelvic pain or pain outside the surgical area are important risk factors for developing chronic pain after hysterectomy [3, 4, 8, 11]. It is not known how the spatial distribution of preexisting pain influences the development of chronic pain, or what role it may play in identifying patients at risk. Thus, it remains necessary to establish a possible association between the spatial spread of pain, its clinical manifestation, and treatment outcome in connection with hysterectomy.

The identification of women at risk of developing chronic pain is of great clinical importance as it allows them to be prepared for what can be expected from surgery. The experimental method of quantitative sensory testing (QST) can quantify the sensitivity to different painful stimulus modalities such as heat, cold and pressure [12–14]. Associations between pain thresholds and development of chronic postoperative pain have been identified and the cold pain threshold was recently found to be associated with maximum pain intensity postoperatively along with consumption of non-opioid analgesics after hysterectomy [15–18].

The definition of persistent postsurgical pain has varied over time [19]. The latest definition from the International Association for Study of Pain includes both pain that was not present before surgery and pain that was present before surgery but increased in intensity [20]. In gynecological clinical practice, however, it is important to distinguish between new pain after surgery, i.e., de novo pain, and pain that remains from before the surgery because both the etiology and prevalence of these two conditions are different. For clarity, throughout this study, de novo chronic postsurgical pelvic pain will be referred to as de novo pelvic pain (DNPP) and remaining chronic pelvic pain from before the surgery as persistent pelvic pain (PPP).

The purpose of the study was to evaluate changes in quantitatively assessed spatial bodily pain within a year following benign hysterectomy. The primary objective was to determine the prevalence of DNPP. Secondary objectives were to determine the prevalence of PPP, and to evaluate risk factors for DNPP and PPP.

## Material and methods

A prospective longitudinal observational multicenter study was conducted between October 2011 and March 2017 investigating the occurrence of DNPP and PPP in women after hysterectomy on benign indication.

The departments of Obstetrics and Gynecology at the public hospitals Linköping University Hospital, Vrinnevi Hospital in Norrköping, Ryhov County Hospital in Jönköping, Värnamo Hospital in Värnamo, and Höglands Hospital in Eksjö in the southeastern health region of Sweden participated in the study.

Women who participated in the randomized multicenter study, the Post-Hysterectomy-Recovery (POSTHYSTREC) trial, which aimed to determine the effect of different models of nurse-led telephone follow-up contact on postoperative recovery after benign hysterectomy were eligible for the study [21].

The women received verbal and written information about the pain study in connection with the POSTHYSTREC trial information approximately one week prior to surgery. Written informed consent was obtained from all participants before inclusion. The participants were allowed to waive the QST measurements. The inclusion and exclusion criteria for the POSTHYSTREC study have previously been described in detail [21]. Briefly, the inclusion criteria were women between 18 and 60 years of age, scheduled for open abdominal or vaginal hysterectomy on benign indication, and able to speak Swedish fluently. One ovary had to be left behind after the operation. Exclusion criteria were concomitant urogynecological surgery, physical disability, severe mental disorder, current drug or alcohol abuse, or expecting more extensive concomitant surgery than hysterectomy, salpingectomy, ovarian resection or appendectomy.

All participating clinics routinely used the perioperative enhanced recovery after surgery (ERAS) program. The mode of anesthesia followed ERAS principles and preferably included intrathecal morphine analgesia alone or in combination with general anesthesia.

### Collection of clinical data

Demographic and clinical data were collected upon entry into the study. Postoperative complications were classified according to Clavien-Dindo [22].

### Pain assessment

**Fig. 1:**
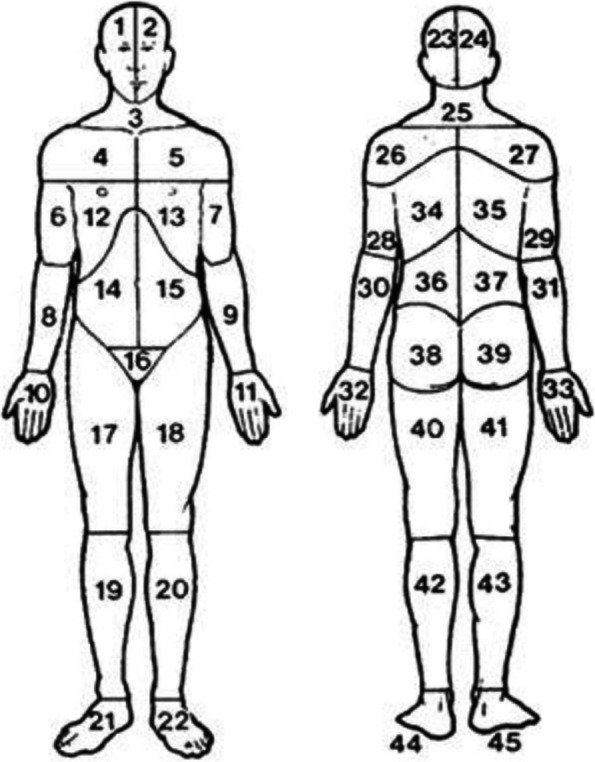
The Margolis pain drawing template [23]. Reprinted with permission from the publisher Wolters Kluwer Health, Inc

Pain was assessed preoperatively, postoperatively for two days, and one year after the hysterectomy using a self-reported questionnaire consisting of simple questions about bodily pain. The detailed questions concerned the average intensity of preoperative pain indicated on a numeric rating scale from 0 (no pain) to 10 (worst imaginable pain), the frequency of occurrence of bodily pain (none, rarely, sometimes, often, almost always/always), the maximum postoperative pain intensity experienced on the day of surgery (day 0) and on the next day (day 1), reported on a numeric scale rating from 0 (no pain) to 6 (very severe pain) indicating the severity of postoperative pain, and the spatial spread of bodily pain drawn on a Margolis’ pain map (Fig. 1). The pain map outlines the spread of pain in areas of the front and back of the body in a total of 45 areas [23]. These were divided into nine regional areas: head (areas 1,2,23,24), neck and shoulders (areas 3–5,25–27), chest (areas 12,13), thoracic back (areas 34,35), abdomen (areas 14,15), pelvis (16), lower back (areas 36–39), upper extremities (areas 6–11,28–33), and lower extremities (areas 17–22,40–45). In order to assess pelvic pain specifically, the spread of pain areas was categorized into four groups: ‘*No pain areas’*, ‘*Pelvis only’*, ‘*Pelvis and other areas’*, and ‘*Non-pelvic areas only’*.

### Hospital Anxiety and Depression Scale (HADS)

The widely used and validated self-rating HADS questionnaire was completed once preoperatively [24, 25]. The form consists of seven questions related to anxiety (the HADS-A subscale) and seven concerning depression (the HADS-D subscale). Each question is scored between 0 and 3. The sum score of each of the subscales is placed into one of three categories: normal (sum score ≤ 8), borderline (sum score > 8 but < 11), and abnormal, indicating severe symptoms (sum score ≥ 11).

### Stress-Coping Inventory (SCI)

To evaluate the stress-coping capability, the SCI form, a validated self-report instrument, was used [26–28]. The form was filled in once preoperatively. It consists of a description of 41 stressful situations. Responses to each item are given using a six-point Likert-type scale, namely: 1-almost never; 2-rarely; 3-occasionally; 4-rather often; 5-very often; 6-almost always. The sum of all scores constitutes a measure of the stress-coping capacity. The cut-off level for low stress coping capacity was set at a score ≤ 169 [27, 28].

### Health-related quality of life (HRQoL)

HRQoL was evaluated by two widely used validated generic quality of life instruments, the EQ-5D-3L [29, 30] and the 36-item Short-Form Health Survey (SF-36) [31, 32]. The EQ-5D-3L health index and the physical component summary (PCS) as well as the mental component summary (MCS) scores from the SF-36 were used to assess HRQoL. A higher index or scores indicated better HRQoL. The forms were filled in preoperatively.

### Quantitative Sensory Testing (QST)

Thermal and pressure pain thresholds were measured using the methods previously described by Lukas et al. [18]. utilizing the Medoc TSA II Neuro Sensory Analyzer (Medoc Ltd. 1 Ha’dekel St. Ramat Yishai 30,095 Israel) for the thermal pain thresholds and a handheld electronic digital algometer (Somedic SenseLab AB, Sösdala, Sweden) for measuring the pressure pain threshold (PPT). Thermal thresholds for the first perceived sensation of pain for cold (CPT) and heat (HPT) were assessed by computerized thermal testing by increasing or decreasing the temperature at a preset rate of change of 1.5°C/s from the baseline temperature of 32°C to 50°C or to 0°C, respectively. By pressing a handheld button connected to the thermo-testing equipment on the first perception of pain the participants registered the pain threshold.

The probe (1 cm^2^ in area) of the algometer was pressed against the skin in a standardized manner with a constant increase in pressure at a rate of approximately 40 kPa/s. The participants were instructed to say “stop” at the first sensation of pain and the concurrent pressure value was registered as the PPT.

### Ethics

The study was approved by the Regional Ethical Board in Linköping (Dnr. 2011/106–31; date of approval May 23; 2011), complies with the Declaration of Helsinki, and is registered with ClinicalTrial.gov (NCT01526668).

### Statistics

Data analyses were performed using the statistical software TIBCO Statistica, version 13.5, (TIBCO Software Inc, Palo Alto CA). Continuous and categorical data are presented as mean (standard deviation) and number (percent), respectively. Continuous variables were analyzed by means of Kruskal–Wallis analysis of variance with subsequent multiple comparisons of mean ranks post-hoc tests or Mann–Whitney U-test, as appropriate. Categorical data were compared using Pearson’s $\chi$ ^2^ test or Fisher’s exact tests, as appropriate. Two-tailed tests were applied, and the level of significance was set at *p* < 0.05.

Binominal logistic regression was used to assess risk factors. In the multivariable models, adjustments were made simultaneously for age, body mass index (BMI), preoperative use of analgesics, mode of hysterectomy, Clavien-Dindo categorization of postoperative complications, and HADS-A and -D scores. The outcome of univariate logistic regression is presented as odds ratio (OR) and 95% confidence interval (95%CI), and correspondingly, adjusted OR (aOR) and 95%CI for the multivariable models.

## Results

**Fig. 2:**
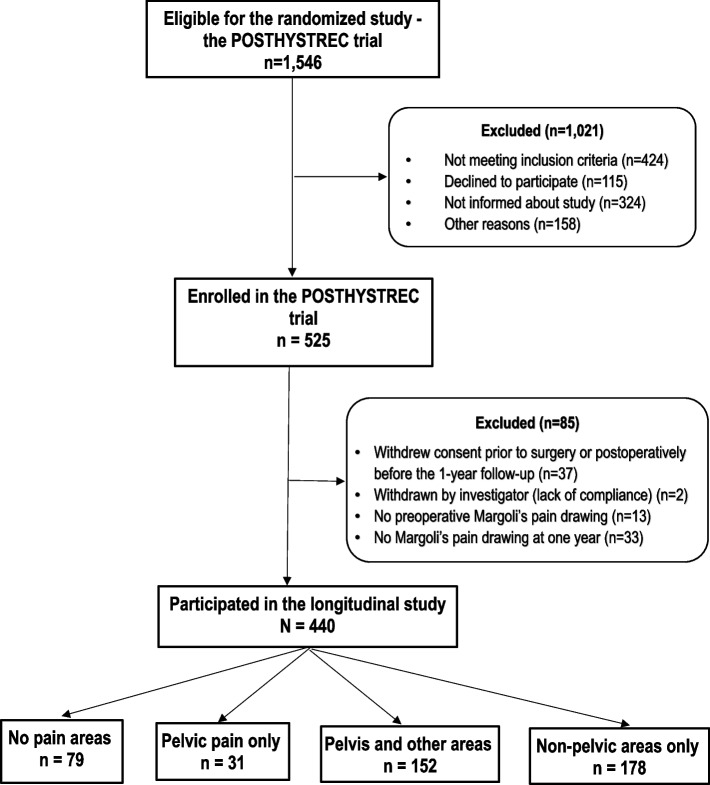
Flow chart of the participants in the longitudinal study of pain spread

The flow chart (Fig. 2) provides an overview of the selection of the 440 women who made up the study population.

### Spatial bodily pain distribution and relation to demographic and clinical characteristics

Of the 440 women, 79 (18.0%) reported ‘*No pain areas’* preoperatively, 31 (7.0%) pain in the ‘*Pelvis only’*, 152 (34.5%) pain in ‘*Pelvis and other areas’*, and 178 (40.5%) pain in ‘*Non-pelvic areas only’* Thus, in total, 183 (41.6%) reported pain involving the pelvis preoperatively.

The demographic and clinical data, subdivided into the four groups according to the spatial spread of pain, are shown in Table 1.. The groups differed significantly in age and preoperative use of analgesics, which were mainly used by women with pain in other areas. According to the post-hoc tests the difference in age between the groups was mainly seen between ‘*No pain areas’* vs. ‘*Pelvis and other areas’* (*p* = 0.01) and ‘*Pelvis and other areas’* vs. ‘*Non-pelvic areas only’* (*p* = 0.01). The number of pain areas reported in the body mapping was significantly higher in women with pain in ‘*Pelvis and other areas’* compared to women with pain in ‘*Non-pelvic areas only’* (8.2 (7.5) vs. 6.9 (6.2), *p* < 0.01). However, by excluding the pelvic area contribution in ‘*Pelvis and other areas’*, the number of other areas did not differ between these two groups (*p* = 0.23). The distribution of the frequency of occurrence of pain also differed between the four groups, with significantly higher frequencies of pain (often or almost always/ always) in the two groups with pain in other areas compared to the pelvis only group. The average intensity of preoperative pain also differed similarly between the groups, mainly attributed to the differences between the ‘*Pelvis only’* group vs. the ‘*Pelvis and other areas’* group (*p* = 0.03) and between ‘*Pelvis and other areas’* vs. ‘*Non-pelvic areas only’* (*p* = 0.01), respectively. The highest preoperative average pain intensity was found in women with ‘*Pelvis and other areas’* (5.0 (2.0)) and the lowest in women with pain in the ‘*Pelvis only’* (3.9 (2.1)).

**Table 1:** Preoperative demographic and clinical data of 440 women undergoing hysterectomy, categorized by patient-reported pain spread

			Patient-reported pain areas preoperatively				
		All (N=440)	No pain areas (n=79)	Pelvis only (n=31)	Pelvis and other areas (n=152)	Non-pelvic areas only (n=178)	*p-value*^†^
*Preoperative variables*							
Age (years)		46.5 (5.5)	47.6 (5.4)	46.5 (4.5)	45.3 (5.4)	47.2 (5.6)	<0.01 ^#^
Body mass index (kg/m^2^)		26.9 (4.6)	26.3 (4.7)	26.8 (4.3)	26.9 (4.9)	27.0 (4.4)	0.71 ^#^
Parous		381 (6.6)	71 (89.9)	30 (96.8)	128 (84.2)	152 (85.4)	0.23
	*Missing data*	*3 (0.7)*	*0 (0.0)*	*0 (0.0)*	*0 (0.0)*	*3 (1.7)*	
Smoking		45 (10.2)	3 (3.8)	3 (9.7)	20 (13.2)	19 (10.7)	0.17
	*Missing data*	*10 (2.3)*	*3 (3.8)*	*0 (0.0)*	*4 (2.6)*	*3 (1.7)*	
ASA physical status classification	Class I	285 (64.8)	53 (67.1)	19 (61.3)	104 (68.4)	109 (61.2)	0.63
	Class II	143 (32.5)	25 (31.6)	12 (38.7)	43 (28.3)	63 (35.4)	
	Class III	12 (2.7)	1 (1.3)	0 (0.0)	5 (3.3)	6 (3.4)	
Comorbidity	Cardiovascular	66 (15.0)	17 (21.5)	3 (9.7)	19 (12.5)	27 (15.2	0.25
	Pulmonary	41 (9.3)	6 (7.6)	0 (0.0)	14 (9.2)	21 (11.8)	0.19
Medication	Analgesics	77 (17.5)	4 (5.1)	0 (0.0)	40 (26.3)	33 (18.5)	<0.0001
	Anti-depressives or sedatives	62 (14.1)	5 (6.3)	3 (9.7)	21 (13.8)	33 (18.5)	0.06
	Hypnotics	19 (14.3)	4 (5.1)	0 (0.0)	6 (3.9)	9 (5.0)	0.62
Physical workload	Sedentary	119 (27.0)	22 (27.8)	11 (35.5)	39 (27.7)	47 (26.4)	0.16
	Medium	122 (27.7)	32 (40.5)	5 (16.1)	41 (27.0)	44 (24.7)	
	Heavy	178 (40.5)	25 (31.7)	14 (45.2)	62 (40.8)	77 (43.3)	
	*Missing data*	*21 (4.8)*	*0 (0.0)*	*1 (3.2)*	*10 (6.6)*	*10 (5.6)*	
Gainfully employed		412 (93.6)	77 (97.5)	31 (100.0)	140 (92.1)	164 (92.1)	0.15
Previous laparotomy		150 (34.1)	29 (36.7)	9 (29.0)	56 (36.8)	56 (31.5)	0.71
	*Missing data*	*4 (0.9)*	*0 (0.0)*	*0 (0.0)*	*0 (0.0)*	*4 (2.2)*	
Indication for hysterectomy	Bleeding disorder	109 (24.8)	15 (19.0)	7 (22.6)	43 (28.3)	44 (24.7)	0.08
	Myoma	203 (46.1)	40 (50.6)	17 (54.8)	62 (40.8)	84 (47.2)	
	Myoma and bleeding	53 (12.0)	10 (12.7)	4 (12.9)	16 (10.5)	23 (12.9)	
	Cervical dysplasia	46 (10.5)	12 (15.2)	1 (3.2)	13 (8.6)	20 (11.2)	
	Pain	27 (6.1)	1 (1.3)	2 (6.4)	18 (11.8)	6 (3.4)	
	Others	2 (0.5)	1 (1.3)	0 (0.0)	0 (0.0*)*	1 (0.6)	
Number of pain areas on body-mapping		5.7 (6.7)	NA	1.0 (--)	8.2 (7.5)	6.9 (6.2)	<0.01*
How often do you have pain	No pain	68 (15.5)	68 (86.0)	0 (0.0)	0 (0.0)	0 (0.0)	<0.0001
	Rarely	33 (7.5)	4 (5.1)	1 (3.2)	15 (9.9)	13 (7.3)	
	Sometimes	136 (30.9)	1 (1.3)	20 (64.5)	47 (30.9)	68 (38.2)	
	Often	111 (25.2)	0 (0.0)	6 (19.4)	51 (33.5)	54 (30.3)	
	Almost always/ always	74 (16.8)	0 (0.0)	0 (0.0)	36 (23.7)	38 (21.4)	
	*Missing data*	*18 (4.1)*	*6 (7.6)*	*4 (12.9)*	*3 (2.0)*	*5 (2.8)*	
Average intensity of preoperative pain (VAS scale 1-10)		(N = 428) 3.8 (2.6)	(n = 79) NA	(n = 27) 3.9 (2.1)	(n = 152) 5.0 (2.0)	(n = 170) 4.4 (2.0)	<0.01 ^#^
HADS-A score		4.9 (4.0)	3.9 (3.7)	3.2 (3.2)	5.4 (4.1)	5.5 (4.1)	<0.01 ^#^
HADS-A (in categories)	Normal	323 (73.4)	66 (83.6)	26 (83.9)	100 (65.8)	131 (73.6)	0.04
	Borderline abnormal	68 (15.5)	8 (10.1)	4 (12.9)	33 (21.7)	23 (12.9)	
	Abnormal	49 (11.1)	5 (6.3)	1 (3.2)	19 (12.5)	24 (13.5)	
HADS-D score		2.6 (3.0)	1.7 (2.3)	1.4 (1.9)	3.0 (3.0)	2.9 (3.4)	<0.001 ^#^
HADS-D (in categories)	Normal	400 (90.9)	76 (96.2)	31 (100.0)	138 (90.8)	155 (87.1)	0.13
	Borderline abnormal	28 (6.4)	2 (2.5)	0 (0.0)	11 (7.2)	15 (8.4)	
	Abnormal	12 (2.7)	1 (1.3)	0 (0.0)	3 (2.0)	8 (4.5)	
SCI score		185.4 (25.5)	186.4 (26.3)	193.4 (21.4)	184.2 (23.1)	184.5 (27.5)	0.33 ^#^
SCI (in categories)	Low stress coping	109 (24.8)	15 (19.0)	5 (16.1)	34 (22.4)	55 (30.9)	0.08
High stress coping		331 (75.2)	64 (81.0)	26 (83.9)	118 (77.6)	123 (69.1)	
SF-36 PCS score		47.9 (9.3)	53.5 (5.5)	50.5 (6.7)	44.7 (10.0)	47.7 (9.1)	<0.0001 ^#^
SF-36 MCS score		47.6 (10.3)	50.4 (7.7)	50.7 (8.7)	36.6 (10.5)	46.7 (11.0)	<0.01 ^#^
EQ-5D-3L health index		0.80 (0.20)	0.90 (0.18)	0.84 (0.18)	0.74 (0.21)	0.80 (0.19)	<0.0001 ^#^
Heat pain threshold (ºC)		(N = 370) 47.5 (2.6)	(n=69) 47.3 (2.6)	(n=25) 48.0 (2.2)	(n=118) 47.2 (2.9)	(n=158) 47.7 (2.5)	0.44 ^#^
Cold pain threshold (ºC)		(N = 370) 3.7 (6.4)	(n=69) 2.6 (5.1)	(n=25) 3.4 (5.6)	(n=118) 5.3 (7.5)	(n=158) 3.1 (5.9)	0.02 ^#^
Pressure pain threshold (kPa)		(N = 367) 503 (199)	(n=69) 515 (220)	(n=25) 530 (225)	(n=116) 476 (193)	(n=158) 513 (189)	0.40 ^#^
*Intra- and postoperative variables*							
Mode of hysterectomy	Total abdominal hysterectomy	310 (70.4)	58 (73.4)	22 (71.0)	109 (71.7)	121 (68.0)	0.86
	Subtotal abdominal hysterectomy	35 (8.0)	7 (8.9)	1 (3.2)	11 (7.2)	16 (9.0)	
	Vaginal hysterectomy	95 (21.6)	14 (17.7)	8 (25.8)	32 (21.1)	41 (23.0)	
Mode of anesthesia	General anesthesia (GA)	162 (36.8)	30 (38.0)	9 (29.0)	59 (38.2)	64 (36.0)	0.14
	Spinal anesthesia + intrathecal morphine	164 (37.3)	30 (38.0)	17 (54.9)	60 (39.5)	57 (32.0)	
	Intrathecal morphine + GA	114 (25.9)	19 (24.0)	5 (16.1)	33 (21.7)	57 (32.0)	
Incision in abdominal wall	No abdominal incision	95 (21.6)	14 (17.7)	8 (25.8)	32 (21.0)	41 (23.0)	0.63
	Low transverse	313 (71.1)	58 (73.4)	22 (71.0)	110 (72.4)	123 (69.1)	
	Midline	25 (5.7)	7 (8.9)	0 (0.0)	8 (5.3)	10 (5.6)	
	*Missing data*	*7 (1.6)*	*0 (0.0)*	*1 (3.2)*	*2 (1.3)*	*4 (2.3)*	
Operation time (minutes)		91 (48)	93 (45)	100 (114)	89 (36)	91 (39)	0.83 ^#^
Estimated bleeding intraoperatively (mL)		177 (218)	184 (225)	138 (131)	178 (180)	180 (255)	0.44 ^#^
Uterus weight (gram)		366 (346)	412 (408)	344 (255)	339 (295)	373 (370)	0.88 ^#^
Blood transfusion (no. of women)		15 (3.4)	2 (2.5)	1 (3.2)	3 (2.0)	9 (5.1)	0.46
Duration of hospital stay (days)		1.8 (1.1)	1.6 (0.8)	1.6 (1.8)	1.8 (1.2)	1.8 (1.0)	0.02 ^#^
Maximum pain intensity day 0 ^§^ (scale 0-6)		(n=432) 3.3 (1.7)	(n=77) 3.1 (1.8)	(n=31) 2.9 (1.8)	(n=148) 3.6 (1.6)	(n=176) 3.3 (1.7)	0.07^#^
Maximum pain intensity day 1 ^§^ (scale 0-6)		(n=432) 3.3 (1.4)	(n=78) 2.8 (1.4)	(n=31) 3.4 (1.4)	(n=148) 3.6 (1.4)	(n=176) 3.2 (1.4)	<0.001^#^
Classification of surgical complications	C-D grade 0	316 (71.8)	61 (77.2)	23 (74.2)	102 (67.1)	130 (73.0)	0.82
	C-D grade I	46 (10.5)	5 (6.3)	4 (12.9)	19 (12.5)	18 (10.1)	
	C-D grade II	64 (14.5)	10 (12.7)	4 (12.9)	26 (17.1)	24 (13.5)	
	C-D grade III	14 (3.2)	3 (3.8)	0 (0.0)	5 (3.3)	6 (3.4)	

Figures denote mean and (standard deviation), or number and (percent)

*ASA* American Society of Anesthesiologists, *C-D *Contracted Clavien-Dindo classification of postoperative complications within six weeks, *EQ-5D-3L *European Quality of Life 5 Dimensions 3 Level version, *HADS-A *Hospital Anxiety and Depression Scale – Anxiety, *HADS-D *Hospital Anxiety and Depression Scale – Depression, *SCI *Stress Coping Inventory

† Continuous data are analyzed by means of non-parametric tests and nominal data by means of Pearson’s chi-squared tests. Missing data are excluded in the statistical analyses

# Kruskal-Wallis analysis of variance

* Mann-Whitney U-test for comparison between ‘*Pelvis and other areas’*and ‘*Non-pelvic areas only’*

§ Day 0 and day 1 indicate day of surgery and the day after surgery, respectively

The psychometric measures HADS-A and D, but not SCI, along with the HRQoL measures EQ-5D-3L, SF-36’s PCS and MCS all revealed significant differences between the groups. The post-hoc tests showed that the differences in HADS-A and HADS-D scores were mainly attributed to differences between ‘*No pain areas’* vs. ‘*Pelvis and other areas’* (*p* = 0.04 and *p* = < 0.01, respectively) and between ‘*Pelvis only’* vs. ‘*Pelvis and other areas’* (*p* = 0.03 and *p* = 0.02, respectively) with lower scores for the former group in both scenarios. For EQ-5D-3L and SF-36’s PCS the pattern of the post-hoc tests was almost identical, with contributions from differences between ‘*No pain areas’* vs. ‘*Pelvis and other areas’* (*p* < 0.0001 and *p* < 0.0001, respectively), between ‘*No pain areas’* vs. ‘*Non-pelvic areas only’* (*p* < 0.0001 and *p* < 0.0001, respectively), and between ‘*Pelvis and other areas’* vs. ‘*Non-pelvic areas only’* (*p* = 0.03 and *p* = 0.03, respectively). In addition, the SF-36’s PCS also differed between ‘*Pelvis only’* and ‘*Pelvis and other areas’* (*p* = 0.01). Concerning the SF-36’s MCS, the main contribution to the significant difference between the groups was, according to the post-hoc test, the difference between ‘*No pain areas’* vs. ‘*Pelvis and other areas’* (*p* = 0.04). All comparisons concerning EQ-5D-3L and the SF-36’ PCS and MCS showed higher scores for the former group in the scenarios except for the comparison between ‘*Pelvis and other areas’* and ‘*Non-pelvic areas only’* where the highest scores were found in the latter group.

Of the three experimental pain threshold measures, only CPT differed significantly between the groups. However, this could not be attributed to differences between any of the subgroups in the post-hoc tests.

The duration of hospital stays differed significantly, albeit modestly, between the groups, mainly because of a difference between the groups ‘*Pelvis only’* and ‘*Non-pelvic areas only’* (*p* = 0.04). In addition, the reported maximum pain intensity on day 1, but not on day 0 also differed significantly between the groups, mainly attributed to the difference between ‘*No pain areas’* and ‘*Pelvis and other areas’* (*p* < 0.001) and between ‘*Pelvis and other areas’’* and ‘*Non-pelvic areas only’* (*p* = 0.02).

### Relation between spatial bodily pain frequency preoperatively and one year postoperatively

The pain frequency preoperatively and one year postoperatively in relation to the four categories of spatial spread of pain is presented in Table 2. Preoperatively, of those with pain in the ‘*Pelvis only’* 26 of 27 (96.3%) had pain sometimes or more often, not significantly different from the corresponding rate for the women with ‘*Pelvis and other areas’* and ‘*Non-pelvic areas only’*, 294 of 322 (91.2%), (*p* = 0.71, Fisher’s exact test). The corresponding figures after one year were three of four (75.0%) and 200 of 227 (88.1%), (*p* = 0.41, Fisher’s exact test).

**Table 2:** Pain frequency in relation to grouping of spatial spread of pain preoperatively (A), and one year postoperatively (B)

**A**	Grouping of spatial spread of pain preoperatively			
Frequency of pain	No pain areas (*n* = 79)	Pelvis only (*n* = 31)	Pelvis and other areas (*n* = 152)	Non-pelvic areas only (*n* = 178)
No pain	68 (86.1)	0 (0.0)	0 (0.0)	0 (0.0)
Rarely	4 (5.1)	1 (3.2)	15 (9.9)	13 (7.3)
Sometimes	1 (1.2)	20 (64.5)	47 (30.9)	68 (38.2)
Often	0 (0.0)	6 (19.4)	51 (33.5)	54 (30.3)
Almost always/always	0 (0.0)	0 (0.0)	36 (23.7)	38 (21.3)
*Missing data*	*6 (7.6)*	*4 (12.9)*	*3 (2.0)*	*5 (2.8)*
**B**	Grouping of spatial spread of pain one year postoperatively			
Frequency of pain	No pain areas (*n* = 208)	Pelvis only (*n* = 5)	Pelvis and other areas (*n* = 41)	Non-pelvic areas only (*n* = 186)
No pain	201 (96.6)	0 (0.0)	0 (0.0)	0 (0.0)
Rarely	6 (2.9)	1 (20.0)	7 (17.1)	20 (10.8)
Sometimes	0 (0.0)	2 (40.0)	7 (17.1)	66 (35.5)
Often	0 (0.0)	0 (0.0)	18 (43.9)	54 (29.0)
Almost always/always	1 (0.5)	1 (20.0)	9 (21.9)	46 (24.7)
*Missing data*	*0 (0.0)*	*1 (20.0)*	*0 (0.0)*	*0 (0.0)*

Figures denote number and (percent)

### Incidence of DNPP and PPP

The change in spatial pain from preoperatively to one year after the hysterectomy is reported in Table 3. DNPP was reported in 6.2% (16/257) of the women and exclusively in the group ‘*Non-pelvic areas only’* Of these, 93.8% (15/16) still had the pain in the other pain areas and one (6.3%) had resolution of the pain in the other pain areas but developed pain in the pelvis as the only location. Pelvic pain disappeared in 83.6% (153/183) and consequently persisted in 16.4% (30/183). No difference was seen in the resolution of pelvic pain between those with pain in the ‘*Pelvis only’* (74.2% (23/31)) and those with pain in the ‘*Pelvis and other areas’* (82.2% (125/152); *p* = 0.30, Pearson’s $\chi$ ^2^ test). None of the women who reported ‘*No pain areas’* preoperatively developed pelvic pain while 27.8% (22/79) reported pain in ‘*Non-pelvic areas only’*, spatially widely spread but predominantly with pain in the abdomen, lower extremities, low back, and head (data not shown), and 72.2% (57/79) still did not report pain areas one year after the surgery. Women with pain in the two groups ‘*Pelvis and other areas’* and ‘*Non-pelvic areas only’’* reported resolution of all pain areas one year after the hysterectomy in 38.8% (128/330), equally distributed between the two groups. However, pain in the other areas, independent of pelvic involvement, was maintained one year after hysterectomy in 60.0% (198/330).

**Table 3:** Association between grouping of spatial spread of pain preoperatively and one year postoperatively

		One year after the hysterectomy			
		No pain areas	Pelvis only	Pelvis and other areas	Non-pelvic areas only
Preoperatively	No pain areas (*n* = 79)	57 (72.2%)	0 (0%)	0 (0%)	22 (27.8%)
	Pelvis only (*n* = 31)	23 (74.2%)	1 (3.2%)	2 (6.5%)	5 (16.1%)
	Pelvis and other areas (*n* = 152)	64 (42.1%)	3 (2.0%)	24 (15.8%)	61 (40.1%)
	Non-pelvic areas only (*n* = 178)	64 (36.0%)	1 (0.5%)	15 (8.4%)	98 (55.1%)

Figures denote number and (percent)

### Risk factors for DNPP

The duration of hospital stay, number of pain areas, intensity of average pain preoperatively, and the regional pain areas of the neck and shoulder, lower back, and upper and lower extremities were independent risk factors for DNPP along with the EQ-5D-3L health index, HADS-A score and HADS-A categories (Table 4).

**Table 4:** Demographic and clinical data of 257 women without pelvic pain undergoing hysterectomy in relation to *de novo* pelvic pain one year post-surgery

		*De novo* pelvic pain		Logistic regression *		
		Yes (n=16)	No (n=241)	Univariate OR (95% CI)	Multivariable † aOR (95% CI)	*p*-value
Preoperative variables						
Age (years)		45.8 (7.8)	47.4 (5.3)	0.95 (0.87-1.04)	0.97 (0.88-1.06)	0.47
Body mass index (kg/m^2^)		27.5 (5.0)	26.8 (4.5)	1.03 (0.93-1.15)	1.03 (0.92-1.16)	0.59
Parous		12 (75.0)	211 (87.6)	0.38 (0.12-1.28)	0.30 (0.08-1.14)	0.08
	*Missing data*	*0 (0.0)*	*3 (1.2)*			
Smoking		2 (12.5)	20 (8.3)	1.54 (0.33-7.24)	1.50 (0.30-7.53)	0.63
	*Missing data*	*0 (0.0)*	*6 (2.5)*			
ASA physical status classification I	I	7 (43.8)	155 (64.3)	1.00 (reference)	1.00 (reference)	
	II-III	9 (56.2)	86 (35.7)	2.32 (0.83-6.44)	2.90 (0.92-9.09)	0.07
Comorbidity	Cardiovascular	3 (18.9)	41 (17.0)	1.13 (0.31-4.13)	1.31 (0.31-5.55)	0.72
	Pulmonary	4 (25.0)	23 (9.5)	3.16 (0.94-10.60)	2.89 (0.79-10.54)	0.12
Medication	Analgesics	2 (12.5)	35 (14.5)	0.84 (0.28-3.86)	0.68 (0.13-3.39)	0.63
	Anti-depressives or sedatives	5 (31.3)	33 (13.7)	2.87 (0.94-8.77)	1.68 (0.46-6.16)	0.43
	Hypnotics	1 (6.3)	12 (5.0)	1.27 (0.15-10.75)	0.44 (0.04-4.72)	0.49
Physical workload	Sedentary	4 (25.0)	65 (27.0)	1.00 (reference)	1.00 (reference)	
	Medium	4 (25.0)	72 (29.9)	0.90 (0.22-3.76)	0.92 (0.21-4.10)	0.91
	Heavy	8 (50.0)	94 (39.0)	1.38 (0.40-4.78)	1.30 (0.35-4.78)	0.70
	*Missing data*	*0 (0.0)*	*10 (4.1)*			
Gainfully employed		15 (93.8)	226 (93.8)	1.00 (0.12-8.05)	3.20 (0.28-37.16)	0.35
Previous laparotomy		2 (12.5)	83 (34.4))	0.27 (0.06-1.19)	0.24 (0.05-1.13)	0.07
	*Missing data*	*0 (0.0)*	*4 (1.7)*			
Indication for hysterectomy	Bleeding disorder	7 (43.8)	52 (21.6)	1.00 (reference)	1.00 (reference)	
	Myoma	5 (31.2)	119 (49.4)	0.31 (0.09-1.03)	0.25 (0.65-0.93)	0.04
	Myoma and bleeding	1 (6.2)	32 (13.3)	0.23 (0.03-1.98)	0.20 (0.02-1.91)	0.16
	Cervical dysplasia	2 (12.5)	30 (12.4)	0.50 (0.10-2.54)	0.60 (0.11-3.28)	0.55
	Pain	1 (6.3)	6 (2.5)	1.24 (0.13-11.86)	0.93 (0.08-10.52)	0.96
	Other	0 (0.0)	2 (0.8)	NA	NA	
Number of pain areas on body-mapping		9.8 (5.6)	4.4 (6.0)	1.09 (1.03-1.16)	1.15 (1.05-1.27)	<0.01
How often did you have pain preoperatively	No pain	0 (0.0)	68 (28.2)	p=0.06‡	NA	
	Rarely	2 (12.5)	15 (6.2)	2.17 (0.36-12.95)	2.54 (0.38-16.95)	0.34
	Sometimes	4 (25.0)	65 (27.0)	1.00 (reference)	1.00 (reference)	
	Often	3 (18.7)	51 (21.2)	0.96 (0.20-4.46)	0.91 (0.17-4.83)	0.91
	Almost always/ always	5 (31.3)	33 (13.7)	2.46 (0.62-9.79)	2.59 (0.53-12.63)	0.24
	*Missing data*	*2 (12.5)*	*9 (3.7)*			
Average intensity of preoperative pain (VAS scale 1-10)		(n =14) 4.6 (2.8)	(n = 6) 2.9 (2.6)	1.27 (1.04-1.55)	1.28 (1.01-1.62)	0.04
Regional pain areas	Head	6 (37.5)	60 (24.9)	1.81 (0.64-5.19)	1.59 (0.50-5.01)	0.43
	Neck and shoulder	11 (68.8)	72 (29.9)	5.16 (1.73-15.40)	4.66 (1.46-14.87)	<0.01
	Chest	1 (6.3)	8 (3.3)	1.94 (0.23-16.56)	1.65 (0.17-16.26)	0.67
	Abdomen	6 (37.5)	67 (27.8)	1.56 (0.54-4.46)	1.39 (0.46-4.21)	0.57
	Thoracic back	3 (18.8)	19 (7.9)	2.70 (0.71-10.30)	2.88 (0.66-12.58)	0.16
	Lower back	11 (68.8)	79 (32.8)	4.51 (1.52-13.43)	4.24 (1.37-13.17)	0.01
	Upper extremities	6 (37.5)	39 (16.2)	3.11 (1.07-9.05)	4.24 (1.18-15.30)	0.03
	Lower extremities	9 (56.3)	54 (22.4)	4.45 (1.58-12.51)	5.23 (1.63-16.79)	<0.01
HADS-A score		7.3 (4.9)	4.7 (4.0)	1.15 (1.03-1.29)	1.10 (0.93-1.29)	0.27
HADS-A (in categories)	Normal	7 (43.8)	190 (78.8)	1.00 (reference)	1.00 (reference)	
	Borderline abnormal	4 (25.0)	27 (11.2)	4.02 (1.10-14.65	4.94 (1.20-20.28)	0.03
	Abnormal	5 (21.2)	24 (10.0)	5.65 (1.66-19.22)	10.61 (1.84-61.03)	<0.01
HADS-D score		4.3 (3.1)	2.4 (3.1)	1.16 (1.02-1.31)	1.06 (0.88-1.29)	0.53
HADS-D (in categories)	Normal	14 (87.5)	217 (90.1)	1.00 (reference)	1.00 (reference)	
	Borderline abnormal	1 (6.2)	16 (6.6)	0.97 (0.12-7.84)	0.25 (0.02-3.11)	0.28
	Abnormal	1 (6.2)	8 (3.3)	1.94 (0.23-16.60)	0.54 (0.04-7.15)	0.64
SCI score		177.8 (32.5)	185.6 (26.7)	0.99 (0.97-1.01)	1.01 (0.98-1.03)	0.56
SCI (in categories)	Low stress coping	7 (43.8)	63 (26.1)	2.20 (0.79-6.15)	0.95 (0.25-3.63)	0.94
	High stress coping	9 (56.2)	178 (73.9)	1.00 (reference)	1.00 (reference)	
SF-36 PCS		44.8 (13.7)	49.8 (8.1)	0.94 (0.90-0.99)	0.95 (0.89-1.00)	0.06
SF-36 MCS		42.7 (10.9)	48.2 (10.1)	0.96 (0.92-0.99)	0.99 (0.93-1.06)	0.79
EQ-5D-3L health index		0.63 (0.26)	0.84 (0.18)	0.03 (0.00-0.19)	0.02 (0.00-0.32)	<0.01
Heat pain threshold (ºC)		(n = 14) 47.7 (2.1)	(n = 213) 47.6 (2.5)	1.01 (0.81-1.26)	1.06 (0.83-1.37)	0.62
Cold pain threshold (ºC)		(n = 14) 3.4 (6.3)	(n = 213) 2.9 (5.6)	1.01 (0.93-1.11)	1.01 (0.92-1.12)	0.78
Pressure pain threshold (kPa)		(n = 14) 470 (188)	(n = 212) 517 (199)	1.00 (1.00-1.00)	1.00 (1.00-1.00)	0.41
*Intra- and postoperative variables*						
Mode of hysterectomy	Total abdominal	10 (62.5)	169 (70.1)	1.00 (reference)	1.00 (reference)	
	Subtotal abdominal	2 (12.5)	21 (8.7)	1.61 (0.33-7.85)	1.51 (0.28-8.24)	0.63
	Vaginal	4 (25.0)	51 (21.2)	1.33 (0.40-4.41)	1.06 (0.30-3.77)	0.93
Mode of anesthesia	GA	8 (50.0)	86 (35.7)	1.00 (reference)	1.00 (reference)	
	SA + IM	4 (25.0)	83 (34.4)	0.52 (0.15-1.79)	0.60 (0.16-2.28)	0.46
	GA + IM	4 (25.0)	72 (29.9)	0.60 (0.17-2.06)	0.67 (0.18-2.47)	0.54
Incision in abdominal wall**	Low transverse	12 (75.0)	169 (71.3)	1.00 (reference)	1.00 (reference)	
	Midline	0 (0.0)	17 (7.2)	p=0.60‡	NA	
Operation time (minutes)		89.6 (33.4)	91.6 (41.9)	1.00 (0.99-1.01)	1.00 (0.98-1.01)	0.89
Estimated bleeding intraoperatively (mL)		157 (162)	182 (250)	1.00 (1.00-1.00)	1.00 (1.00-1.00)	0.67
Uterus weight (gram)		387 (456)	384 (377)	1.00 (1.00-1.00)	1.00 (1.00-1.00)	0.88
Blood transfusion (no. of women)		1 (6.2)	10 (4.1)	1.54 (0.18-12.84)	2.12 (0.16-28.30)	0.57
Duration of hospital stay (days)		2.3 (0.9)	1.7 (0.9)	1.40 (0.98-1.99)	1.50 (1.02-2.21)	0.04
Maximum pain intensity day 0 ^§^ (scale 0-6)		(n=16) 3.4 (1.4)	(n=237) 3.2 (1.7)	1.08 (0.80-1.46)	1.03 (0.74-1.42)	0.79
Maximum pain intensity day 1 ^§^ (scale 0-6)		(n=16) 3.6 (1.2)	(n=238) 3.00 (1.4)	1.33 (0.92-1.94)	1.36 (0.89-2.06	0.15
Classification of surgical complications	C-D grade 0	12 (74.0)	179 (74.3)	1.00 (reference)	1.00 (reference)	
	C-D grade I	1 (6.2)	22 (9.1)	0.67 (0.08-5.41)	0.63 (0.07-5.59)	0.68
	C-D grades II-III	3 (18.8)	40 (16.6)	1.11 (0.30-4.10)	1.03 (0.27-3.98)	0.97

*aOR* adjusted odds ratio, *C-D* Clavien-Dindo classification of postoperative complications, *CI* confidence interval, *EQ-5D-3L* Euroqol form-five dimensions-three levels, *GA* general anesthesia, *HADS-A* Hospital Anxiety and Depression Scale- Anxiety, *HADS-D* Hospital Anxiety and Depression Scale- Depression, *IM* intrathecal morphine, *MCS*, mental component summary, *NA* not applicable, *PCS *physical component summary, *SA* spinal anesthesia, *SCI* Stress Coping Inventory, *SF-36* Short Form 36 items

*Missing data not included in the analyses

† Adjusted for age, Body mass index, smoking, preoperative use of analgesics, mode of hysterectomy, postoperative complications (Clavien-Dindo), HADS-A, and HADS-D

**Includes abdominal hysterectomies only

‡ Because of a cell with no observations, the logistic regression could not be calculated. Instead, the p-value of Fisher’s exact test or Pearson chi-square test is given, as appropriate

§ Day 0 and day 1 indicate day of surgery and the day after surgery, respectively

### Risk factors for PPP

Age was an independent risk factor for PPP. For each year that age increased, the risk decreased by 11%. In addition, the number of pain areas, the regional pain area of the lower extremities, and the frequency of occurrence of pain preoperatively were independent risk factors (Table 5).

**Table 5:** Demographic and clinical data of 183 women with pelvic pain undergoing hysterectomy in relation to persistent pelvic pain one year post-surgery

		Persistent pelvic pain		Logistic regression *		
		Yes (n=30)	No (n=153)	Univariate OR (95% CI)	Multivariable † aOR (95% CI)	*p*-value
*Preoperative variables*						
Age (years)		42.9 (5.5)	46.0 (5.1)	0.88 (0.82-0.96)	0.89 (0.81-0.97)	<0.01
Body mass index (kg/m^2^)		28.2 (5.2)	26.6 (4.7)	1.06 (0.99-1.15)	1.08 (0.99-1.17)	0.09
Parous		26 (86.7)	132 (86.3)	1.03 (0.33-3.26)	2.22 (0.46-10.59)	0.32
Smoking		4 (13.3)	19 (12.4)	1.16 (0.36-3.70)	1.69 (0.48-6.02)	0.41
	*Missing data*	*2 (6.7)*	*2 (1.3)*			
ASA physical status classification	I	23 (76.7)	100 (65.4)	1.00 (reference)	1.00 (reference)	
	II-III	7 (23.3)	53 (34.6)	0.57 (0.23-1.43)	0.43 (0.14-1.26)	0.12
Comorbidity	Cardiovascular	2 (6.7)	20 (13.2)	0.48 (0.10-2.15)	0.48 (0.10-2.36)	0.36
	Pulmonary	4 (13.3)	10 (6.5)	2.20 (0.64-7.55)	2.85 (0.76-10.75)	0.12
Medication	Analgesics	9 (30.0)	31 (20.3)	1.69 (0.70-4.04)	1.17 (0.41-3-35)	0.76
	Anti-depressives or sedatives	4 (13.3)	20 (13.1)	1.02 (0.32-3.24)	0.76 (0.20-2.89)	0.68
	Hypnotics	1 (3.3)	5 (3.3)	1.02 (0.11-9.06)	NA	
Physical workload	Sedentary	10 (33.3)	40 (26.1)	1.00 (reference)	1.00 (reference)	
	Medium	5 (16.7)	41 (26.8)	0.49 (0.15-1.55)	0.53 (0.15-1.82)	0.31
	Heavy	11 (36.7)	65 (42.5)	0.68 (0.26-1.74)	0.67 (0.23-1.92)	0.46
	*Missing data*	*4 (13.3)*	*7 (4.6)*			
Gainfully employed		26 (86.7)	145 (94.8)	2.79 (0.78-9.94)	0.32 (0.07-1.52)	0.15
Previous laparotomy		11 (36.7)	54 (35.3)	0.94 (0.42-2.12)	0.96 (0.40-2.33)	0.93
Indication for hysterectomy	Bleeding disorder	6 (20.0)	44 (28.8)	1.00 (reference)	1.00 (reference)	
	Myoma	13 (43.3)	66 (43.1)	1.44 (0.51-4.09)	1.29 (0.39-4.28)	0.68
	Myoma and bleeding	1 (3.3)	19 (12.4)	0.39 (0.04-3.43)	0.35 (0.04-3.34)	0.36
	Cervical dysplasia	3 (10.0)	11 (7.2)	2.00 (0.43-9.29)	2.49 (0.48-12.84)	0.28
	Pain	7 (23.3)	13 (8.5)	3.95 (1.13-13.83)	3.28 (0.81-13.25)	0.10
Number of pain areas on body-mapping		11.2 (12.0)	4.7 (2.1)	1.35 (1.08-1.67)	1.08 (1.02-1.14)	<0.01
Regional pain areas	Head	10 (33.3)	37 (24.2)	1.57 (0.67-3.65)	1.65 (0.65-4.16)	0.29
	Neck and shoulder	12 (40.0)	46 (30.1)	1..55 (0.69-3.48)	1.32 (0.53-3.30)	0.55
	Chest	0 (0.0)	5 (3.3)	p=0.59 ‡	NA	
	Abdomen	18 (60.0)	71 (46.4)	1.73 (0.78-3.84)	1.39 (0.60-3.33)	0.46
	Thoracic back	2 (6.7)	17 (11.1)	0.57 (0.12-2.61)	0.48 (0.10-2.31)	0.36
	Lower back	16 (53.3)	69 (45.1)	1.39 (0.63-3.05)	1.30 (0.52-3.25)	0.58
	Upper extremities	7 (23.3)	25 (16.3)	1.56 (0.60-4.02)	1.15 (0.35-3.76)	0.81
	Lower extremities	15 (50.0)	41 (26.8)	2.73 (1.23-6.08)	3.14 (1.25-7.86)	0.01
How often did you have pain preoperatively	Rarely	0 (0.0)	16 (10.5)	NA	NA	
	Sometimes	2 (6.7)	65 (42.5)	1.00 (reference)	1.00 (reference)	
	Often	16 (53.3)	41 (26.8)	12.69 (2.77-58.05)	12.27 (2.44-61.80)	<0.01
	Almost always/always	11 (36.7)	25 (16.3)	14.30 (2.96-69.13)	12.75 (2.24-72.66)	<0.01
	*Missing data*	*1 (3.3)*	*6 (3.9)*			
Average intensity of preoperative pain (VAS scale 1-10)		5.8 (1.7)	4.7 (2.1)	1.07 (1.02-1.12)	1.24 (0.98-1.58)	0.08
	*Missing data*	*1 (3.3)*	*3 (2.0)*			
HADS-A score		6.2 (4.3)	4.8 (4.0)	1.09 (0.99-1.19)	1.08 (0.95-1.22)	0.25
HADS-A (in categories)	Normal	15 (53.3)	110 (71.9)	1.00 (reference)	1.00 (reference)	
	Borderline abnormal	10 (33.3)	27 (17.6)	2.55 (1.04-6.23)	1.94 (0.72-5.24)	0.19
	Abnormal	4 (13.3)	16 (10.5)	1.72 (0.51-5.79)	2.14 (0.54-8.41)	0.28
HADS-D score		3.4 (2.9)	2.6 (2.9)	1.09 (0.96-1.23)	1.00 (0.84-1.20)	0.96
HADS-D (in categories)	Normal	28 (93.3)	141 (92.1)	1.00 (reference)	1.00 (reference)	
	Borderline abnormal	2(6.7)	9 (5.9)	1.12 (0.23-5,46)	0.76 (0.13-4.44)	0.76
	Abnormal	0 (0.0)	3 (2.0)	NA	NA	
SCI score		179.3 (23.7)	187.1 (22.7)	0.99 (0.97-1.00)	0.98 (0.96-1.01)	0.15
SCI (in categories)	Low stress coping	10 (33.3)	29 (19.0)	2.14 (0.90-5.05)	2.51 (0.87-7.26)	0.09
	High stress coping	20 (66.7)	124 (81.0)	1.00 (reference)	1.00 (reference)	
SF-36 PCS		43.3 (10.4)	46.1 (9.6)	0.97 (0.94-1.01)	0.98 (0.93-1.03)	0.39
SF-36 MCS		44.6 (11.9)	47.8 (9.9)	0.97 (0.94-1.01)	0.99 (0.94-1.04)	0.71
EQ-5D-3L health index		0.69 (0.23)	0.77 (0.20)	0.20 (0.04-1.08)	0.38 (0.04-3.30)	0.38
Heat pain threshold (ºC)		(n = 21) 46.6 (3.4)	(n = 122) 47.5 (2.7)	0.91 (0.78-1.05)	0.93 (0.79-1.10)	0.42
Cold pain threshold (ºC)		(n = 21) 6.1 (8.5)	(n = 122) 4.7 (7.06)	1.02 (0.96-1.09)	1.02 (0.95-1.09)	0.66
Pressure pain threshold (kPa)		(n = 21) 465 (210)	(n = 120) 489 (198)	1.00 (1.00-1.00)	1.00 (1.00-1.00)	0.46
*Intra- and postoperative variables:*						
Mode of hysterectomy	Total abdominal	21 (70.0)	110 (71.9)	1.00 (reference)	1.00 (reference)	
	Subtotal abdominal	4 (13.3)	8 (5.2)	2.62 (0.72-3.49)	3.96 (0.89-17.56)	0.07
	Vaginal	5 (16.7)	35 (22.9)	0.74 (0.26-2.13)	0.59 (0.19-1.87)	0.44
Mode of anesthesia	GA	11 (36.7)	57 (37.3)	1.00 (reference)	1.00 (reference)	
	SA + IM	11 (36.7)	66 (43.1)	0.96 (0.35-2.14)	1.83 (0.62-5.35)	0.27
	GA + IM	8 (26.6)	30 (19.6)	1.38 (0.50-3.80)	2.13 (0.69-6.64)	0.19
Incision in abdominal wall**	Low transverse	23 (92.0)	109 (92.4)	1.00 (reference)	1.00 (reference)	
	Midline	1 (4,0)	7 (5.9)	0.68 (0.08-5.77)	0.49 (0.04-5.54)	0.56
	*Missing data*	*1 (4.0)*	*2 (1,7)*			
Operation time (minutes)		89.1 (32.5)	91.0 (60.4)	1.00 (0.99-1.01)	1.00 (0.99-1.01)	0.46
Estimated bleeding intraoperatively (mL)		186 (188)	168 (171)	1.00 (1.00-1.00)	1.00 (1.00-1.00)	0.57
Uterus weight (gram)		256 (255)	356 (292)	1.00 (1.00-1.00)	1.00 (1.00-1.00)	0.06
Blood transfusion (no. of women)		0 (0.0)	4 (2.6)	p=1.00 ‡	NA	
Duration of hospital stay (days)		2.1 (2.0)	1.7 (1.2)	1.19 (0.95-1.50)	1.15 (0.89-1.49)	0..29
Maximum pain intensity day 0 ^§^ (scale 0-6)		(n=30) 4.1 (1.5)	(n=149) 3.3 (1.7)	1.41 (1.07-1.85)	1.26 (0.95-1.68)	0.11
Maximum pain intensity day 1 ^§^ (scale 0-6)		(n=30) 3.7 (1.6)	(n=149) 3.6 (1.4)	1.06 (0.80-1.41)	0.97 (0.70-1.33)	0.72
Classification of surgical complications	C-D grade 0	18 (60.0)	107 (69.9)	1.00 (reference)	1.00 (reference)	
	C-D grade I	6 (20.0)	17 (11.1)	2.10 (0.73-6.03)	1.52 (0.44-5.21)	0.50
	C-D grades II-III	6 (20.0)	29 (19.0)	1.23 (0.45-3.38)	0.62 (0.19-2.10)	0.44

*aOR* adjusted odds ratio, *C-D* Clavien- Dindo classification of postoperative complications, *CI* confidence interval, *EQ-5D-3L* EuroQol form-five dimensions-three levels, *GA* general anesthesia, *HADS-A* Hospital Anxiety and Depression Scale - Anxiety *HADS-D* Hospital Anxiety and Depression Scale - Depression, *IM* intrathecal morphine,* MCS* mental component summary, *PCS* physical component summary, *SA* spinal anesthesia, *SCI* Stress Coping Inventory, *SF-36* Short Form 36 items

*Missing data not included in the analyses

† Adjusted for age, Body -mass index, smoking, preoperative use of analgesics, mode of hysterectomy, postoperative complications (Clavien-Dindo), HADS-A, and HADS-D

** Includes abdominal hysterectomies only

‡ Because of a cell with 0 observations, the logistic regression could not be calculated. Instead, the p-value of Fisher’s exact test is given

§ Day 0 and day 1 indicate day of surgery and the day after surgery, respectively

## Discussion

The study revealed that 6.2% of the women developed DNPP and 16.4% had PPP one year after the hysterectomy. DNPP developed exclusively in women with pain in other areas preoperatively. Over 80% of the women who reported pelvic pain preoperatively achieved complete resolution at the one-year follow-up, ranging from 82% in women who besides pain in the pelvis had pain in other areas, to 90% with preoperative pelvic pain only. Preoperative risk factors for DNPP were identified, including preoperative pain intensity, the number of pain areas, the pain areas neck and shoulders, lower back, and upper and lower extremities along with low quality of life and anxiety. The risk factors for PPP were almost the same but also included a higher frequency of preoperative pain and younger age.

The prevalence of DNPP one year after hysterectomy was in line with the recently published study from the Swedish National Quality Registry for Gynecological Surgery (GynOp) where Grundström et al. found *DNPP* in 7.8% [33]. The data collection in the GynOp was prospective, as in the present study. A prospective American multicenter study reported a *DNPP* rate of 3.6% [34]. Brandsborg et al. reported a Danish nationwide postal questionnaire study where DNPP was found in 14.9% [9]. The data in that study were collected retrospectively more than a year after the surgery and there was a significant risk of recall bias. A Dutch study with a similar design to the present one reported a 9.0% prevalence of chronic postsurgical pain one year after hysterectomy [8]. However, the pain in that study was not distinctly related to the pelvis but was described as mainly originating from the lower abdomen. Another study reported persistent postsurgical pelvic pain four months after hysterectomy in 26.1% of the women [6]. These studies highlight the difficulty of comparing results and emphasize the importance of using a uniform design and time indication after surgery, a uniform definition of pain, and delineation of the area of pain.

The prevalence of PPP reported by 16.4% of the respondents in this study corresponded to the rate reported in previous studies [9, 33, 35, 36].

The reported rate of resolution of chronic pelvic pain after hysterectomy varies between 76 and 88% and our result falls within these limits [3, 33–38]. Thus, consistent with other studies, this study indicated that hysterectomy is successful in the treatment of chronic pelvic pain, irrespective of whether that is pelvic pain alone or combined with pain elsewhere in the body. However, the resolution of pelvic pain did not appear to affect the resolution of pain outside the pelvis.

To our knowledge this is the first study to evaluate associations between preoperative pain body mapping and DNPP and PPP one year after surgery. Only a few studies in benign gynecology have reported associations between pain elsewhere and persistent postsurgical pain but without specifying the location or the number of painful areas or distinguishing between DNPP and PPP [6, 7, 9]. A recent systematic review and meta-analysis of the few published studies on the spatial spread of pain and persistent postsurgical pain after hysterectomy reported that patients with preoperative pain elsewhere had a three-fold higher risk of developing persistent postsurgical pain [4].

The present study showed that the risk of DNPP and PPP was significantly associated with an increasing number of pain areas and location of the pain elsewhere. This may indicate that individuals who developed DNPP or PPP already carried a state of aberrant neuro-modulation that may have been triggered to accelerate potential mechanisms involved in the development or maintenance of these pain conditions. Such an association has been shown in fertile-aged women with chronic pain conditions caused by endometriosis [39–41]. Central sensitization probably contributes to chronic pain development in both DNPP and PPP patients, although through different mechanisms. A preoperative pain condition such as widespread pain suggests an established central sensitization that, due to supraspinal mechanisms, facilitates DNPP development [42]. Conversely, central sensitization in PPP patients is caused by excitatory synaptic modulation in the dorsal horn of the spinal cord due to noxious stimuli through peripheral nerves from persistent pelvic nociceptive pain. As a result, the excitatory state of the dorsal horn continues even after the noxious stimulus, such as hysterectomy, is eliminated [43]. Viscerosomatic convergence may also be a major contributor to PPP, further amplifying pain transmission in the spinal cord and perception in higher brain centers [44]. None of the women without preoperative pain developed DNPP, suggesting a rather low risk in those women due to the absence of pain conditions and a state of central sensitization. However, this interpretation should be made with great caution.

Severe acute postoperative pain has been repeatedly identified as a risk factor for the development of chronic pain after hysterectomy [2, 3, 5, 6, 11, 43]. The present study found an association between preoperative pelvic pain and maximum pain intensity on postoperative day 1 but we could not confirm an association between acute postoperative pain and PPP or DNPP.

There is a growing body of literature indicating the association between psychological characteristics and postsurgical pain [45]. While Han et al. and Pinto et al. showed that preoperative anxiety was a risk factor for persistent postsurgical pain they did not discriminate between de novo pain and persistent preoperative pain although the effect of anxiety on these conditions may be different [5, 7]. The latter was supported by our findings that anxiety was a risk factor only for *DNPP* but not for PPP. Han et al. and Benolo et al. found likewise that depression was a risk factor for persistent post-hysterectomy pain [2, 5]. However, as with anxiety, they did not differentiate persistent postsurgical pain into de novo and persistent preoperative pain. This may explain why we did not find associations between depression and *DNPP* or PPP.

Information on the relationship between the mode of hysterectomy and persistent postsurgical pain is equivocal. While Pinto et al. reported an association between abdominal hysterectomy and the development of chronic postsurgical pain [6], others did not find such an association [3, 9, 37]. We found no association between surgical mode and DNPP or PPP one year postoperatively.

Chronic pain generally has a negative impact on HRQoL, and patients with multiple pain locations are usually the most severely affected [46]. This seemed consistent with our results. The perceived HRQoL was lowest when pelvic pain co-occurred with pain in other areas. This might imply that pelvic pain contributed to a greater extent to lower HRQoL when it occurred simultaneously with pain in other areas of the body. The measures of HRQoL appeared to predict DNPP, but not PPP. A low EQ-5D-3L health index preoperatively was a risk factor for DNPP one year after the hysterectomy but not for PPP, suggesting a multifactorial etiology of postsurgical DNPP. The group of women with PPP consisted mainly of women who preoperatively had widespread bodily pain including pelvic pain. These women even had the lowest EQ-5D health index preoperatively.

Consistent with other studies, younger age, anxiety, pain elsewhere, and preoperative pain frequency were risk factors for PPP [2, 5–9, 11]. The relationship between preoperative QST and persistent postsurgical pain has been repeatedly investigated, with conflicting results [15–18, 47]. A recent systematic review concluded that no consistency was found for a single QST parameter having a predictive role for the development of chronic postoperative pain [14]. The present study seemed to support that conclusion.

The relationship between preoperative QST and postoperative persistent pain has been extensively researched, with conflicting results [16, 17]. Although some studies have shown that thermal and pressure pain thresholds were predictors of high postoperative pain intensity and persistent pain [18, 47], a systematic review came to the opposite conclusion [15]. Our study failed to demonstrate associations between pain thresholds and PPP.

### Strength and limitations

The study has several strengths including the prospective, longitudinal multicenter design, the large number of participants, and the use of an ERAS protocol according to the best standard of care, along with the use of validated forms and methods. In addition, the indications for benign hysterectomy were quite similar to those presented in the GynOp indicating that the study population was representative of the Swedish population [48]. Thus, the result may be generalized at least to communities or countries with similar populations and healthcare facilities.

The study has limitations. It may suffer from selection bias. Anxiety, depression, or fear of experimental pain may have been reasons for refraining from participating in the study. Reluctance and a potential apprehension about participation in the section of the study concerning measurement of pain thresholds was evident with more than 15% refraining from participation in the pain threshold measurements. Moreover, the questions concerning the self-reported pain measures were not strictly validated. However, the questions that were asked were simple in their construction and unambiguous, which should mean a low risk of misinterpretation. Another limitation may be the use of pain frequency as a measure of chronic pain instead of the more commonly used definition of a pain duration of > 3 months. In addition, the information on pain intensity or frequency of pain was not related to the individual areas of pain but represented an overall measure of the condition.

## Conclusion

The risk of DNPP after hysterectomy was not negligible, affecting one in 16, but seemed to affect exclusively those who had pain conditions in other parts of the body preoperatively. More than 80% of women with pelvic pain were cured. Women at risk for DNPP and PPP after hysterectomy could be identified preoperatively. Information about the risk factors should be included in the preoperative counseling before benign hysterectomy.

## Data Availability

The datasets generated and/or analyzed during the current study are not publicly available due to study participant privacy but are available from the corresponding author (Peter.Lukas@liu.se) on reasonable request and in accordance with Swedish legislation.

## Abbreviations

aOR: Adjusted Odds Ratio
BMI: Body Mass Index
CPT: Cold Pain Threshold
DNPP: *De Novo* Persistent Pain
GynOp: The Swedish National Quality Registry for Gynecological Surgery
HADS: Hospital Anxiety and Depression Scale
HPT: Heat Pain Threshold
HRQoL: Health-Related Quality of Life
MCS: Mental Component Summary
OR: Odds Ratio
PCS: Physical Component Summary
PPP: Persistent Pelvic Pain
PPT: Pressure Pain Threshold
SCI: Stress Coping Inventory
SF-36: Short From 36 Health Survey
QST: Quantitative Sensory Testing

## References

[1] Brandsborg B, Nikolajsen L, Kehlet H, Jensen TS. Chronic pain after hysterectomy. Acta Anaesthesiol Scand. 2008;52(3):327–31. 10.1111/j.1399-6576.2007.01552.x. 18269384 10.1111/j.1399-6576.2007.01552.x

[2] Benlolo S, Hanlon JG, Shirreff L, Lefebvre G, Husslein H. Predictors of persistent postsurgical pain after hysterectomy-A prospective cohort study. J Minim Invasive Gynecol. 2021;28(12):2036–2046.e1. 10.1016/j.jmig.2021.05.017. 34077793 10.1016/j.jmig.2021.05.017

[3] Pokkinen SM, Nieminen K, Yli-Hankala A, Kalliomäki ML. Persistent posthysterectomy pain: A prospective, observational study. Eur J Anaesthesiol. 2015;32(10):718–24. 10.1097/EJA.0000000000000318. 26258656 10.1097/EJA.0000000000000318

[4] Sharma LR, Schaldemose EL, Alaverdyan H, Nikolajsen L, Chen D, Bhanvadia S, et al. Perioperative factors associated with persistent postsurgical pain after hysterectomy, cesarean section, prostatectomy, and donor nephrectomy: a systematic review and meta-analysis. Pain. 2022;163(3):425–35. 10.1097/j.pain.0000000000002361. 34121077 10.1097/j.pain.0000000000002361

[5] Han C, Ge Z, Jiang W, Zhao H, Ma T. Incidence and risk factors of chronic pain following hysterectomy among Southern Jiangsu Chinese Women. BMC Anesthesiol. 2017;17(1):103. 10.1186/s12871-017-0394-3. 10.1186/s12871-017-0394-3 PMC5553861 28800726

[6] Pinto PR, McIntyre T, Nogueira-Silva C, Almeida A, Araújo- Soares V, Almeida A, et al. Risk factors for persistent postsurgical pain in women undergoing hysterectomy due to benign causes: a prospective predictive study. J Pain. 2012;13(11):1045–57. 10.1016/j.jpain.2012.07.014. 23063345 10.1016/j.jpain.2012.07.014

[7] Pinto PR, McIntyre T, Araújo-Soares V, et al. Psychological factors predict an unfavorable pain trajectory after hysterectomy: a prospective cohort study on chronic postsurgical pain. Pain. 2018;159(5):956–67. 10.1097/j.pain.0000000000001170. 29419656 10.1097/j.pain.0000000000001170

[8] Theunissen M, Peters ML, Schepers J, Maas JW, Tournois F, van Suijlekom HA, et al. Recovery 3 and 12 months after hysterectomy: epidemiology and predictors of chronic pain, physical functioning, and global surgical recovery. [published correction appears in Medicine (Baltimore). 2017;96(20):e6957; 10.1097/MD.0000000000006957]. Medicine (Baltimore). 2016;95(26):e3980; 10.1097/MD.0000000000003980. 10.1097/MD.0000000000003980 PMC4937912 27367998

[9] Brandsborg B, Nikolajsen L, Hansen CT, Jensen TS. Risk factors for chronic pain after hysterectomy: a nationwide questionnaire and database study. Anesthesiology. 2007;106(5):1003–12. 10.1097/01.anes.0000265161.39932.e8. 17457133 10.1097/01.anes.0000265161.39932.e8

[10] Aasvang EK, Gmaehle E, Hansen JB, Gmaehle B, Forman JL, Schwarz J, et al. Predictive risk factors for persistent postherniotomy pain. Anesthesiology. 2010;112(4):957–69. 10.1097/ALN.0b013e3181d31ff8. 20234307 10.1097/ALN.0b013e3181d31ff8

[11] Brandsborg B, Dueholm M, Nikolajsen L, Kehlet H, Jensen TS. A prospective study of risk factors for pain persisting 4 months after hysterectomy. Clin J Pain. 2009;25(4):263–8. 10.1097/AJP.0b013e31819655ca. 19590472 10.1097/AJP.0b013e31819655ca

[12] Fruhstorfer H, Lindblom U, Schmidt WC. Method for quantitative estimation of thermal thresholds in patients. J Neurol Neurosurg Psychiatry. 1976;39:1071–5. 10.1136/jnnp.39.11.1071. 188989 10.1136/jnnp.39.11.1071 PMC1083305

[13] Rolke R, Baron R, Maier C, Tölle TR, Treede -DR, Beyer A et al. Quantitative sensory testing in the German Research Network on Neuropathic Pain (DFNS): Standardized protocol and reference values. [published correction appears in Pain. 2006;125(1–2):197]. Pain. 2006;123(3):231–243; 10.1016/j.pain.2006.01.041. 10.1016/j.pain.2006.01.041 16697110

[14] Petersen KK, Vaegter HB, Stubhaug A, Wolff A, Scammell BE, Arendt-Nielsen L, et al. The predictive value of quantitative sensory testing: a systematic review on chronic postoperative pain and the analgesic effect of pharmacological therapies in patients with chronic pain. Pain. 2021;162(1):31–44. 10.1097/j.pain.0000000000002019. 32701654 10.1097/j.pain.0000000000002019

[15] Sangesland A, Støren C, Vaegter HB. Are preoperative experimental pain assessments correlated with clinical pain outcomes after surgery? A systematic review Scand J Pain. 2017;15:44–52. 10.1016/j.sjpain.2016.12.002. 28850344 10.1016/j.sjpain.2016.12.002

[16] van Helmond N, Aarts HM, Timmerman H, Olesen SS, Drewes AM, Wilder-Smith OH, et al. Is preoperative quantitative sensory testing related to persistent postsurgical pain? A systematic literature review Anesth Analg. 2020;131(4):1146–55. 10.1213/ANE.0000000000004871. 32925335 10.1213/ANE.0000000000004871

[17] Braun M, Bello C, Riva T, Hönemann C, Doll D, Urman RD et al. Quantitative sensory testing to predict postoperative pain. Curr Pain Headache Rep. Curr Pain Headache Rep. 2021;25(1):3. 10.1007/s11916-020-00920-5. 10.1007/s11916-020-00920-5 PMC7808998 33443676

[18] Lukas P, Gerdle B, Nilsson L, Wodlin NB, Fredrikson M, Arendt-Nielsen L et al. Association between experimental pain thresholds and trajectories of postoperative recovery measures after benign hysterectomy. [published correction appears in J Pain Res. 2023;16:677–679. 10.2147/JPR.S410626]. J Pain Res. 2022;15:3657–3674. Published 2022 Nov 23; 10.2147/JPR.S383795. 10.2147/JPR.S383795 PMC9701515 36447527

[19] Werner MU, Kongsgaard UEI. Defining persistent post-surgical pain: is an update required? Br J Anaesth. 2014;113(1):1–4. 10.1093/bja/aeu012. 24554546 10.1093/bja/aeu012

[20] Schug SA, Lavand’homme P, Barke A, Korwisi B, Rief W, Treede RD. The IASP classification of chronic pain for ICD-11: chronic postsurgical or posttraumatic pain. Pain. 2019;160(1):45–52. 10.1097/j.pain.0000000000001413. 30586070 10.1097/j.pain.0000000000001413

[21] Kassymova G, Sydsjö G, Borendal Wodlin N, Nilsson L, Kjølhede P. The effect of follow-up contact on recovery after benign hysterectomy: a randomized, single-blinded, four-arm, controlled multicenter trial. J Womens Health (Larchmt). 2021;30(6):872–81. 10.1089/jwh.2020.875219. 33232628 10.1089/jwh.2020.8752

[22] Dindo D, Demartines N, Clavien PA. Classification of surgical complications. A new proposal with evaluation in a cohort of 6336 patient and results of a survey. Ann Surg. 2004;240(2):205–213; 10.1097/01.sla.0000133083.54934.ae. 10.1097/01.sla.0000133083.54934.ae PMC1360123 15273542

[23] Margolis RB, Tait RC, Krause SJ. A rating system for use with patient pain drawings. Pain. 1986;24(1):57–65. 10.1016/0304-3959(86)90026-6. 2937007 10.1016/0304-3959(86)90026-6

[24] Zigmond AS, Snaith RP. The Hospital Anxiety and Depression Scale. Acta Psychiatr Scand. 1983;67:361–70. 10.1111/j.1600-0447.1983.tb09716.x. 6880820 10.1111/j.1600-0447.1983.tb09716.x

[25] Bjelland I, Dahl AA, Haug TT, Neckelmann D. The validity of the Hospital Anxiety and Depression Scale. An updated literature review. J Psychosom Res. 2002;52(2):69–77; 10.1016/s0022-3999(01)00296-3. 10.1016/s0022-3999(01)00296-3 11832252

[26] Ryding EL, Wijma B, Wijma K, Rydhström H. Fear of childbirth during pregnancy may increase the risk of emergency cesarean section. Acta Obstet Gynecol Scand. 1998;77(5):542–7. 9654177

[27] Söderqvist J, Wijma B, Wijma K. The longitudinal course of post-traumatic stress after childbirth J Psychosom Obstet Gynaecol. 2006;27(2):113–9. 10.1080/01674820600712172. 16808086 10.1080/01674820600712172

[28] Kjølhede P, Borendal Wodlin N, Nilsson L, Wijma K. Impact of stress coping capacity on recovery from abdominal hysterectomy in a fast-track programme: A prospective longitudinal study. [published correction appears in BJOG. 2012;119(10):1291]. BJOG. 2012;119(8):998–1007; 10.1111/j.1471-0528.2012.03342.x. 10.1111/j.1471-0528.2012.03342.x 22568450

[29] EuroQol Group. EuroQol–a new facility for the measurement of health-related quality of life. Health Policy. 1990;16(3):199–208. 10.1016/0168-8510(90)90421-9. 10109801 10.1016/0168-8510(90)90421-9

[30] Bjurström K, Sun S, Gerdtham UG, Henriksson M, Johannesson M, Levin LÅ, et al. Swedish experience-based value sets for EQ-5D health states. Qual Life Res. 2014;23(2):431–42. 10.1007/s11136-013-0496-4. 23975375 10.1007/s11136-013-0496-4 PMC3967073

[31] McHorney CA, Ware JE Jr, Raczek AE. The MOS 36-Item Short-Form Health Survey (SF-36): II. Psychometric and clinical tests of validity in measuring physical and mental health constructs. Med Care. 1993;31(3):247–263; 10.1097/00005650-199303000-00006. 10.1097/00005650-199303000-00006 8450681

[32] Sullivan M, Karlsson J, Ware JE Jr. The Swedish SF-36 Health Survey--I. Evaluation of data quality, scaling assumptions, reliability and construct validity across general populations in Sweden. Soc Sci Med. 1995;41(10):1349–1358; 10.1016/0277-9536(95)00125-q. 10.1016/0277-9536(95)00125-q 8560302

[33] Grundström H, Fredrikson M, Alehagen S, Berterö C, Kjølhede P. Incidence of self-reported pelvic pain and risk factors for pain 1 year after benign hysterectomy: A register study from the Swedish National Quality Registry for Gynecological Surgery. Acta Obstet Gynecol Scand. 2023;102(10):1359–70. 10.1111/aogs.14455. 36073635 10.1111/aogs.14455 PMC10541156

[34] Kjerulff KH, Langenberg PW, Rhodes JC, Harvey LA, Guzinski GM, Stolley PD. Effectiveness of hysterectomy. Obstet Gynecol. 2000;95(3):319–26. 10.1016/s0029-7844(99)00544-x. 10711536 10.1016/s0029-7844(99)00544-x

[35] Stovall TG, Ling FW, Crawford DA. Hysterectomy for chronic pelvic pain of presumed uterine etiology. Obstet Gynecol. 1990;75:676–9. 2248635

[36] Hillis SD, Marchbanks PA, Peterson HB. The effectiveness hysterectomy for chronic pelvic pain. Obstet Gynecol. 1995;86:941–5. 10.1016/0029-7844(95)00304-a. 7501344 10.1016/0029-7844(95)00304-a

[37] As-Sanie S, Till SR, Schrepf AD, Griffith KC, Tsodikov A, Missmer SA, et al. Incidence and predictors of persistent pelvic pain following hysterectomy in women with chronic pelvic pain. Am J Obstet Gynecol. 2021;225(5):568.e1–568.e11. 10.1016/j.ajog.2021.08.038. 34464585 10.1016/j.ajog.2021.08.038 PMC9297195

[38] Hartmann KE, Ma C, Lamvu GM, Langenberg PW, Steege JF, Kjerulff KH. Quality of life and sexual function after hysterectomy in women with preoperative pain and depression. Obstet Gynecol. 2004;104(4):701–9. 10.1097/01.AOG.0000140684.37428.48. 15458889 10.1097/01.AOG.0000140684.37428.48

[39] Grundström H, Gerdle B, Alehagen S, Berterö C, Arendt-Nielsen L, Kjølhede P. Reduced pain thresholds and signs of sensitization in women with persistent pelvic pain and suspected endometriosis. Acta Obstet Gynecol Scand. 2019;98(3):327–36. 10.1111/aogs.13508.E. PMID: 30472739. 30472739 10.1111/aogs.13508

[40] Raimondo D, Raffone A, Renzulli F, Sanna G, Raspollini A, Bertoldo L, et al. Prevalence and risk factors of central sensitization in women with endometriosis. J Minim Invasive Gynecol. 2023;30(1):73–80.e1. 10.1016/j.jmig.2022.10.007. PMID: 36441085. 36441085 10.1016/j.jmig.2022.10.007

[41] Orr NL, Huang AJ, Liu YD, Noga H, Bedaiwy MA, Williams C, et al. Association of Central Sensitization Inventory Scores with pain outcomes after endometriosis surgery. JAMA Netw Open. 2023;6(2):e230780. 10.1001/jamanetworkopen.2023.0780. PMID:36848090; PMCID:PMC9972194. 36848090 10.1001/jamanetworkopen.2023.0780 PMC9972194

[42] Harte SE, Harris RE, Clauw DJ. The neurobiology of central sensitization. J Appl Biobehav Res. 2018;23:e12137. 10.1111/jabr.12137.

[43] Chapman CR, Vierck CJ. The transition of acute postoperative pain to chronic pain: An integrative overview of research on mechanisms. J Pain. 2017;18(4):359.e1–359.e38. 10.1016/j.jpain.2016.11.004. 27908839 10.1016/j.jpain.2016.11.004

[44] Lamvu G, Carrillo J, Ouyang C, Rapkin A. Chronic Pelvic Pain in Women: A Review. JAMA. 2021;325(23):2381–91. 10.1001/jama.2021.2631. PMID: 34128995. 34128995 10.1001/jama.2021.2631

[45] Bair MJ, Robinson RL, Katon W, Kroenke K. Depression and pain comorbidity: a literature review. Arch Intern Med. 2003;163(20):2433–45. 10.1001/archinte.163.20.2433. 14609780 10.1001/archinte.163.20.2433

[46] Lamé IE, Peters ML, Vlaeyen JW, Kleef Mv, Patijn J. Quality of life in chronic pain is more associated with beliefs about pain, than with pain intensity. Eur J Pain. 2005;9(1):15–24. 10.1016/j.ejpain.2004.02.006 10.1016/j.ejpain.2004.02.006 15629870

[47] Müller M, Bütikofer L, Andersen OK, Heini P, Arendt-Nielsen L, Jüni P, et al. Cold pain hypersensitivity predicts trajectories of pain and disability after low back surgery: a prospective cohort study. Pain. 2021;162(1):184–94. 10.1097/j.pain.0000000000002006. 33035044 10.1097/j.pain.0000000000002006

[48] Ehrström S. Hysterektomi på benign indikation. Årsrapport från GynOp-registret avseende operationer utförda år 2022. [In Swedish]. https://www.gynop.se/wp-content/uploads/2023/05/Arsrapport-Hysterektomi-utford-ar-2022.pdf. [Last accessed: 06/26/2024]

